# The role of dynamic instability in microtubule organization

**DOI:** 10.3389/fpls.2014.00511

**Published:** 2014-10-07

**Authors:** Tetsuya Horio, Takashi Murata

**Affiliations:** ^1^Department of Natural Sciences, Nippon Sport Science UniversityYokohama, Japan; ^2^Division of Evolutionary Biology, National Institute for Basic BiologyOkazaki, Japan; ^3^Department of Basic Biology, School of Life Sciences, The Graduate University for Advanced StudiesOkazaki, Japan

**Keywords:** cortical array, dynamic instability, GTP hydrolysis, microtubule, phragmoplast, tubulin

## Abstract

Microtubules are one of the three major cytoskeletal components in eukaryotic cells. Heterodimers composed of GTP-bound α- and β-tubulin molecules polymerize to form microtubule protofilaments, which associate laterally to form a hollow microtubule. Tubulin has GTPase activity and the GTP molecules associated with β-tubulin molecules are hydrolyzed shortly after being incorporated into the polymerizing microtubules. GTP hydrolysis alters the conformation of the tubulin molecules and drives the dynamic behavior of microtubules. Periods of rapid microtubule polymerization alternate with periods of shrinkage in a process known as dynamic instability. In plants, dynamic instability plays a key role in determining the organization of microtubules into arrays, and these arrays vary throughout the cell cycle. In this review, we describe the mechanisms that regulate microtubule dynamics and underlie dynamic instability, and discuss how dynamic instability may shape microtubule organization in plant cells.

## INTRODUCTION

Microtubules are present in all eukaryotic cells and play important roles in a variety of cellular processes (Dustin, 1984; Bray, 2001). A key characteristic of microtubules is their dynamic nature, and microtubules dynamically alter their organization in response to the needs of the cell. Microtubule arrays in the somatic cells of higher plants undergo dynamic changes in conformation throughout the cell cycle. Specifically, microtubules form a cortical microtubule array, pre-prophase band, mitotic spindle, and phragmoplast during each round of the cell cycle (Wasteneys, 2002).

Each individual microtubule elongates or shrinks to fulfill a specific role, and growing and shrinking microtubules co-exist in the same cytoplasm. For instance, chromosome alignment at the metaphase plate depends on the simultaneous presence of both growing and shrinking microtubules. When chromosomes are moved toward the metaphase plate, the microtubules attached to one side of the kinetochore must elongate, while those at the other side must shrink, even though all of these microtubules exist in the same cellular environment. Furthermore, during anaphase, the microtubules that connect spindle poles to kinetochores must disassemble to pull the chromosomes toward the pole, whereas those that interdigitate at the equatorial plane must remain assembled to maintain the distance between the poles. Thus, mechanisms exist that permit microtubules in different regions of a cell to display distinct dynamic properties. In this review, we discuss the molecular mechanisms that control microtubule dynamics and the effect that these dynamics have on the organization of plant microtubule arrays. For this purpose, we summarize our current understanding of mechanisms of microtubule nucleation, elongation, and shrinkage, and describe how such dynamic properties contribute to the organization of cortical arrays and phragmoplasts.

## MOLECULAR COMPOSITION OF THE MICROTUBULE

**FIGURE 1:**
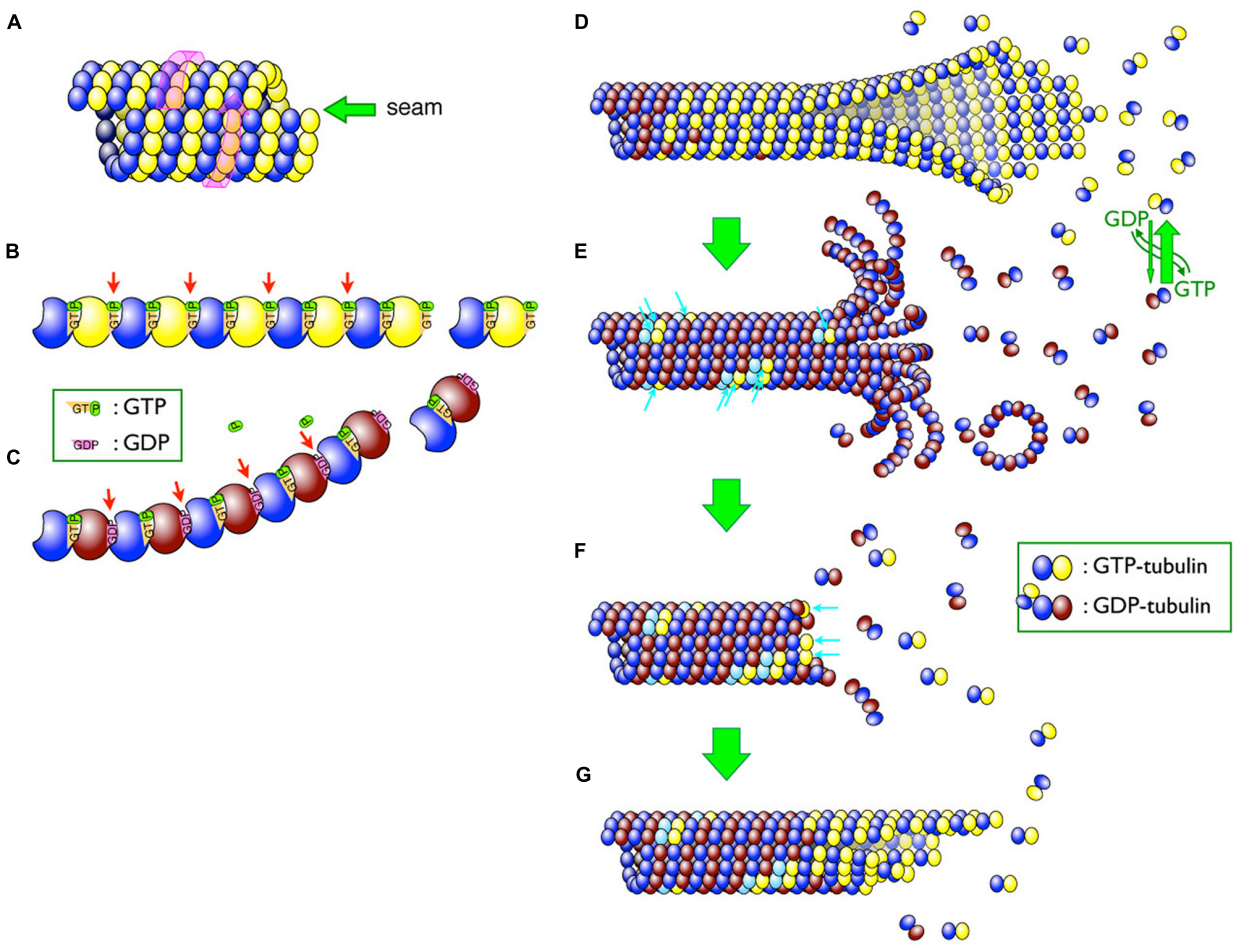
**Schematic diagram of microtubules. (A)** Lateral arrangement of tubulin dimers in the microtubule. The tubulin molecules are laterally aligned along the three-start left-handed helix (indicated by a pink wrap-around arrow) and different tubulin molecules thus meet at a seam (indicated by a green arrow). **(B,C)** GTP hydrolysis causes a conformational change in protofilaments. Whereas GTP–tubulins **(B)** have a straight conformation that fits well in the wall of the microtubule, GDP-tubulins **(C)** tend to curve outward from the microtubule lattice. β-tubulins bound to GDP are shaded brown, whereas those bound to GTP are yellow. Changes in inter-dimer interactions are indicated by arrows. **(D–G)** Depolymerization and rescue of microtubules. While newly incorporated tubulin dimers are all GTP–tubulins **(D)**, GTP molecules are relatively quickly hydrolyzed and most of the dimers in the microtubule become GDP-tubulin (shaded in brown; **D**) Remnant GTP–tubulins in the microtubule are indicated by blue arrows **(E)**. Depolymerization can be stopped by remnant GTP–tubulins as a “rescue” action **(F)**. The addition of new GTP-tubulins (blue arrows in **F**) resumes the growth, and the addition of the tubulin dimers continues **(G)**.

The microtubule is a polymer of α- and β-tubulin dimers (**Figure 1A**). The tubulin dimers assemble in a head-to-tail manner to form linear polymers called protofilaments (**Figure 1B**). Multiple protofilaments, typically 13, assemble into tubular microtubule structures. Since tubulin dimers are aligned in the same orientation in a protofilament and all protofilaments in a microtubule are parallel to each other, the microtubule is intrinsically polar. The end that exposes β-tubulin (Mitchison, 1993; i.e., the plus end) grows more rapidly than the end exposing α-tubulin (i.e., the minus end; Cote and Borisy, 1981). The assembly and disassembly of microtubules occurs by the addition and release of subunits from the ends, respectively. The local concentration of available tubulin subunits is the key factor that determines the rate of microtubule growth/shrinkage. Although microtubule assembly generally depends on the concentration of available subunits, this does not fully explain the dynamic properties of the microtubule. Thus, the mechanisms that control localized microtubule assembly and disassembly have long since been a topic of discussion.

## DYNAMIC INSTABILITY OF MICROTUBULES

The co-existence of growing and shrinking microtubules in the same conditions is termed “dynamic instability.” This phenomenon was first predicted based on observations of fixed *in vitro* reconstituted microtubules (Mitchison and Kirschner, 1984a,b). Because reconstituted microtubules can be generated from purified tubulin, the dynamic behavior of microtubules is considered to be an intrinsic property, and not caused by external controlling factors. The co-existence of growing and shrinking microtubules was verified by observations of individual microtubules using unfixed preparations, and the “rescue” event, i.e., the transition from shrinkage back to growth, was described (Horio and Hotani, 1986; Kirschner and Mitchison, 1986; Walker et al., 1988; van der Vaart et al., 2009). The microtubules formed from purified tubulin dimers polymerize in a concentration- and temperature-dependent manner. However, they depolymerize in a stochastic manner that is unaffected by the concentration of available tubulin or the state of the neighboring microtubules. The execution and timing of rescue events also occur in a stochastic manner (Kirschner and Mitchison, 1986; van der Vaart et al., 2009). Under assembly competent conditions, microtubules remain in the growth state for the majority of the time and all microtubules grow at a similar rate determined by the concentration of tubulin dimers present in the environment. The rate of microtubule depolymerization is several fold faster than that of polymerization and does not directly correlate with the subunit concentration. The transition from a growing to a shrinking state is called “catastrophe.” Catastrophe events appear to occur stochastically, and the depolymerizing microtubules may or may not be rescued and resume growth. The following parameters are frequently used to characterize microtubules in a particular set of conditions: the growth rate and duration, the shortening rate and duration, and the frequency of catastrophe and rescue events. Recent observations of *in vitro* reconstituted microtubules revealed that the molecular events during microtubule polymerization are more complex than simple subunit addition at the ends. The ends of each protofilament of growing microtubules randomly alternates between periods of subunit addition (growth) and loss (shrinkage), and pausing (Kerssemakers et al., 2006; Schek et al., 2007; Gardner et al., 2008). Microtubule growth means that subunits are added more frequently than they are lost.

## STRUCTURAL BASIS OF MICROTUBULE GROWTH AND SHRINKAGE

High-resolution analyses using cryo-electron microscopy revealed the structure of assembling and disassembling microtubules (Mandelkow et al., 1991; Chretien et al., 1995). During assembly, tubulin dimers are added to the end of protofilaments and the protofilaments bind laterally to each other to form a two-dimensional sheet-like structure (**Figure 1D**). The longitudinal edges of the sheet ultimately meet to form a tubular structure.

The guanine nucleotide molecule (i.e., GTP) bound to the β-tubulin molecule plays a key role in dynamic instability. Each α- and β-tubulin molecule binds one molecule of GTP (). The GTP bound to the α-tubulin is neither hydrolyzed nor exchanged, whereas that bound to the β-tubulin can be exchanged when the dimer is free in solution. These exchangeable GTP molecules are located at the head-to-tail interface between subunits along a protofilament (Nogales et al., 1998; Lowe et al., 2001) and are hydrolyzed shortly after being incorporated into the polymer. The hydrolysis of the GTP to GDP affects the conformation of the inter-dimer interface (). Structural studies indicated that, while the protofilament composed of GTP–tubulin is almost straight, the natural conformation of the protofilament of GDP-tubulin is curved outward from the wall of the microtubule (Wang and Nogales, 2005; Nogales and Wang, 2006). Lateral interactions between GTP–tubulin molecules provide the force that holds these molecules together, resisting the natural tendency of protofilaments composed of GDP–tubulins to curve outward. Microtubule disassembly can be triggered by a subtle change in the lateral interactions between the protofilaments. The tubulin subunits near the ends of rapidly growing microtubules are more likely to be bound to GTP (**Figure 1D**), and the loss of the GTP–tubulin portion, known as the GTP-cap, renders the microtubules more prone to depolymerization (**Figure 1E**). This GTP-cap model is supported by experimental evidence and is widely accepted (Carlier et al., 1987; O’Brien et al., 1987; Drechsel and Kirschner, 1994; Caplow and Shanks, 1996).

The mechanism underlying the rescue event is relatively poorly understood (). It was postulated that the addition of new tubulin dimers to the shrinking ends of multiple protofilaments stops depolymerization (Bayley et al., 1990). Direct observations using a GTP–tubulin-specific antibody revealed that GTP–tubulin patches exist in the middle of microtubules (Dimitrov et al., 2008; **Figure 1E**, arrows). These GTP–tubulin patches may contribute to the rescue events following depolymerization. Although the frequency of rescue events is important for controlling microtubule organization *in vivo*, the mechanism underlying GTP–tubulin patch formation is unknown (Dimitrov et al., 2008).

A noteworthy inconsistency of the GTP-cap model is the behavior of the minus, or slower growing end. In principle, the minus end should be more inclined to undergo depolymerization than the quicker growing plus end, because the minus end would be expected to lose its GTP-cap more readily and frequently than the plus end; however, experimental evidence indicates that the opposite is true (Horio and Hotani, 1986; Walker et al., 1988). Furthermore, the newly exposed minus ends created by physical severing of the microtubules are quite stable, as these ends used to be located in the middle of longer microtubules (Tran et al., 1997). By severing a microtubule, a new plus end and minus end are created and the subunits near the newly formed ends are predominantly in the GDP form. According to the GTP-cap model, both new microtubules are highly likely to depolymerize from their newly created ends. However, this is only true for the new plus ends. Although microtubule behavior after cutting is important in living cells, the details of this behavior are unknown.

## REGULATORS OF MICROTUBULE DYNAMICS

Since microtubules formed *in vivo* cannot be permitted to grow and shrink in a stochastic manner, multiple proteins interact with microtubules to control their intrinsic instability. A group of proteins promotes the nucleation of the microtubules. Most *in vivo* nucleation events of microtubules occur at microtubule organizing centers (MTOCs) and are facilitated by the γ-tubulin microtubule nucleator complex (γ-TuC; Job et al., 2003; Kollman et al., 2011). γ-tubulin and its complexed proteins assemble to form an open ring-like structure, which is assumed to serve as a template for the protofilament arrangement, known as the γ-tubulin ring complex (γ-TuRC). One interesting characteristic of the γ-TuRC is that it forces the tubulin to form 13-protofilament microtubules, whereas protofilaments that are allowed to associate spontaneously typically give rise to 14-protofilament microtubules (Evans et al., 1985).

Regulator proteins that control microtubule dynamics can be categorized into two groups, i.e., those that promote the assembly of microtubules and those that work to destabilize microtubules. Different regulators use different mechanisms to regulate microtubule dynamics. Microtubule-associated proteins (MAPs) are a group of microtubule-stabilizing proteins that co-purify with tubulin (Hamada, 2007; van der Vaart et al., 2009; Gardiner, 2013). Conventional MAPs bind to tubulin at multiple binding sites and, by cross-bridging multiple subunits, stabilize inter-subunit interactions. MAPs stabilize microtubules by suppressing dissociation of the subunits, and are known to promote both the nucleation and assembly of microtubules *in vitro*.

One group of MAPs, XMAP215 and its homologous proteins, exhibits unique functions. This group of proteins promotes microtubule nucleation and increases the rate of microtubule assembly; however, unlike other MAPs, members of this group contain a single microtubule-binding site, called a TOG-domain. These proteins preferentially bind to tubulin dimers and processively track the growing plus ends of microtubules (Hamada, 2007; Al-Bassam and Chang, 2011). A detailed description of the interaction between the XMAP215 group of proteins and tubulin is presented in an accompanying paper of this issue (Hamada, 2014).

MAP65/Ase1/PRC1, a MAP conserved in higher eukaryotes, contributes to microtubule bundle formation by crosslinking antiparallel microtubules (Chang-Jie and Sonobe, 1993; Smertenko et al., 2000). Microtubule-bound MAP65 stops microtubule depolymerization during the shrinkage phase (Stoppin-Mellet et al., 2013), as do other MAPs (Ichihara et al., 2001). In addition, MAP65 modifies the mechanical property of microtubules. Microtubules decorated with MAP65-1 or Ase1 become more flexible than MAP-free microtubules (Portran et al., 2013). Softening of microtubules is considered to be necessary for angle-dependent bundle formation, which in turn is essential for organizing microtubules into cortical arrays in plant cells (see below).

A particular group of MAPs, called +TIPs, tracks microtubule plus ends. One of the +TIP proteins, EB1, preferentially binds the seam of microtubules and is thought to stabilize the tubular conformation of microtubules. Several different types of +TIPs exist in the cell. Some +TIP proteins are known to affect the organization of cellular microtubules; however, in many cases, it is unclear whether this is a direct or indirect effect of these proteins (Kumar and Wittmann, 2012). CLASP was the first +TIP member to be identified. Subsequent research revealed that CLASPs are multifunctional proteins, with functions beyond plus-end capping, and use TOG-domains to bind to the microtubules (Kumar and Wittmann, 2012). The function of CLASPs is a little different from that of another TOG-domain protein, MAP215. While MAP215 promotes microtubule assembly, CLASPs stabilize microtubules by promoting rescue events (Al-Bassam et al., 2010; Al-Bassam and Chang, 2011).

Several proteins destabilize microtubules. Typical microtubule-destabilizing proteins act by promoting catastrophe events. Various members of the kinesin family of microtubule motor proteins are known to depolymerize microtubules based on experiments conducted mostly in animal and fungal cells (van der Vaart et al., 2009). Kinesin-13 has a high affinity for depolymerizing curved protofilaments, and the three-dimensional structure of kinesin-13 fits very well to the curved protofilaments of disassembling microtubules. Thus, kinesin-13 is considered to promote microtubule catastrophe by causing protofilaments to adopt a curved conformation (Moores et al., 2002; Newton et al., 2004). Kinesin-8, which glides along microtubules and accumulates at their plus ends, is also known to promote microtubule disassembly from the plus ends (Gupta et al., 2006; Varga et al., 2006; Mayr et al., 2007). Interestingly, kinesin-8 preferentially promotes the disassembly of longer microtubules over shorter ones. This activity is useful for maintaining the length of microtubules within a preferable range *in vivo*.

## REGULATORS OF MICROTUBULE DYNAMICS IN LAND PLANTS

As discussed above, little is known about the regulators of microtubule dynamics in land plants. Genes encoding γ-TuRC proteins are conserved in land plants (Murata et al., 2007), and plant microtubules likely have 13 protofilaments. The ortholog of XMAP215 in *Arabidopsis thaliana* is MOR1 (Whittington et al., 2001), and its temperature-sensitive mutant *mor1-1* shows reduced microtubule dynamicity (Kawamura and Wasteneys, 2008), which is consistent with MAP215 function in animal cells. While the *A. thaliana* genome encodes only one XMAP215 ortholog, it has 9 MAP65 genes (Hussey et al., 2002). Among these genes, *MAP65-1* and *MAP65-2* likely function in microtubule bundling in interphase cells (Lucas et al., 2011). In the case of +TIPs, genes characterized in animal cells are not conserved in land plant genomes, except for EB1 and CLASP. *Arabidopsis thaliana* has three EB1 genes, and EB1c has been demonstrated to stabilize microtubules (Komaki et al., 2010). CLASP was also found to limit catastrophe events during interphase microtubule array organization (Ambrose et al., 2011). Therefore, the roles of these +TIPs in regulating microtubule dynamics in land plants are consistent with those of their animal orthologs.

Kinesin-13 has been demonstrated to mediate microtubule depolymerization in land plants (Oda and Fukuda, 2012). However, the function of kinesins and their localization in the mitotic apparatus do not necessarily correspond to those of homologous kinesins in other organisms (Miki et al., 2014). Therefore, additional research should aim to identify the functions of different groups of kinesins in controlling microtubule dynamics.

In addition to regulators that are conserved in many eukaryotes, land plants also have unique regulators. SPIRAL2 is a land plant-specific microtubule binding protein that reduces pausing of microtubule growth (Yao et al., 2008). In addition, it binds to microtubule crossover sites and recruits the microtubule-severing protein katanin (Wightman et al., 2013). The role of microtubule severing in microtubule organization is a central topic of this review, and is discussed in detail in the following sections.

## HOW IS THE CORTICAL ARRAY ORGANIZED IN PLANT CELLS?

Microtubule organization in land plant cells changes during the cell cycle (Wasteneys, 2002). In interphase, microtubules localize along the plasma membrane as a cortical array. Before entering mitosis, microtubules form a bundle, called the preprophase band, at the future site of cytokinesis. During mitosis, they form an acentrosomal mitotic spindle. During cytokinesis, they form a phragmoplast, which is involved in cell plate deposition. Among these organized structures, the microtubule dynamics in the interphase cortical array have been the most extensively studied during the last decade (Shaw et al., 2003; Chan et al., 2007; Oda and Fukuda, 2012; Lindeboom et al., 2013), probably because microtubules near the cell surface can be more readily visualized than those in the cell interior. Due to the simple two-dimensional organization of cortical microtubule arrays, computer simulation techniques can be used to analyze array organization. In the following section, we discuss the mechanism by which cortical array organization is related to microtubule dynamics.

## MECHANISM OF MICROTUBULE ALIGNMENT BY MICROTUBULE–MICROTUBULE INTERACTIONS

Cortical array organization is divided into two levels. The primary level involves parallel array formation, and the higher-order level involves guiding the parallel array perpendicular to the axis of growth. The primary level of organization is explained by local interactions between microtubules. Dixit and Cyr (2004) found that when the growing plus ends of microtubules encounter the flanks of existing microtubules, the response of the microtubules depends on the angle of contact. When the growing plus ends hit the wall of existing microtubules at a shallow angle (i.e., <40 degrees), the ends change direction and continue to grow in parallel with the existing microtubules, forming a parallel bundle. When they encounter the wall at a large angle (i.e., >40∘), the microtubules run across the existing microtubules or initiate a catastrophe event. Computer simulations suggest that angle-dependent bundling and catastrophe represent an effective mechanism for the local ordering of microtubules (Dixit and Cyr, 2004).

Microtubules are formed on preassembled tubulin seeds called γ-tubulin complexes. Upon nucleation, most of the complexes bind to the existing microtubules (Nakamura et al., 2010) and nucleate new microtubules as 40∘ branches (Murata et al., 2005) or parallel bundles (0∘; Chan et al., 2009), although a minor fraction of the complexes (1.4% in Nakamura et al., 2010) nucleate new microtubules without binding to the existing microtubules (Nakamura et al., 2010; Kirik et al., 2012). The 40∘ branching and a small amount of microtubule-independent nucleation continuously supply discordant microtubules to the ordered microtubule array. To maintain a parallel array, such discordant microtubules must be removed or reoriented. The angle-dependent ordering is an effective mechanism for maintaining parallel arrays of microtubules.

An *A. thaliana* mutant of the *Xenopus* MAP215 ortholog *MOR1, mor1-1,* exhibits defects in forming parallel microtubule arrays (Whittington et al., 2001). This phenotype can be explained by a defect of microtubule–microtubule interactions. Because the MAP215 family of proteins mediates tubulin exchange at the tip of microtubules, both growth and shrinkage of microtubules are suppressed in the *mor1-1* mutant (Kawamura and Wasteneys, 2008). Suppression of microtubule dynamics might limit microtubule interactions. Computer simulations demonstrated that such defects in microtubule dynamics result in the formation of disorganized microtubule arrays (Allard et al., 2010; Eren et al., 2010).

Although angle-dependent microtubule bundling is important for microtubule ordering, the underlying molecular mechanism is unclear. Because microtubule bundling by the microtubule bundling protein MAP65-1 depends on contact angles between microtubules *in vitro* (Tulin et al., 2012), MAP65-1 may mediate microtubule bundling in living cells. However, MAP65-1 specifically bundles antiparallel microtubules (Tulin et al., 2012), whereas almost all microtubules encountered at shallow-angles form bundles in living cells (Dixit and Cyr, 2004). The encountered bundles are likely equal mixes of parallel and antiparallel arrays. In addition, double mutant plants of *map65-1* and *map65-2*, the sister gene of tobacco *MAP65-1* in *A. thaliana*, can form microtubule bundles (Lucas et al., 2011). Other bundling proteins, such as kinesin-5 tetramers, may be involved in angle-dependent bundling, in addition to MAP65s. Interestingly, a mutant of kinesin-5 in *A. thaliana, rsw4* (Bannigan et al., 2007), shows disordered microtubules in cortical arrays (Wiedemeier et al., 2002).

## ROLE OF KATANIN, THE MICROTUBULE-SEVERING PROTEIN

After microtubules nucleate as branches on existing microtubules, the minus ends of microtubules are often severed by the microtubule severing protein katanin (Nakamura et al., 2010). The minus ends formed by severing start to depolymerize (Shaw et al., 2003). Because the plus ends usually elongate, the severed microtubules show treadmilling as a whole and maintain their overall length. The depolymerization of minus ends of the branched microtubules might contribute to the removal of diagonally running microtubules in the array.

The mechanism underlying minus-end depolymerization after cutting is unknown. It is assumed that microtubule ends created by cutting start to depolymerize because of a lack of GTP caps. However, minus ends created by artificial cutting *in vitro* do not depolymerize, although the plus ends do (Tran et al., 1997). Therefore, we expect that an uncharacterized mechanism exists that depolymerizes minus ends in living cells.

**FIGURE 2:**
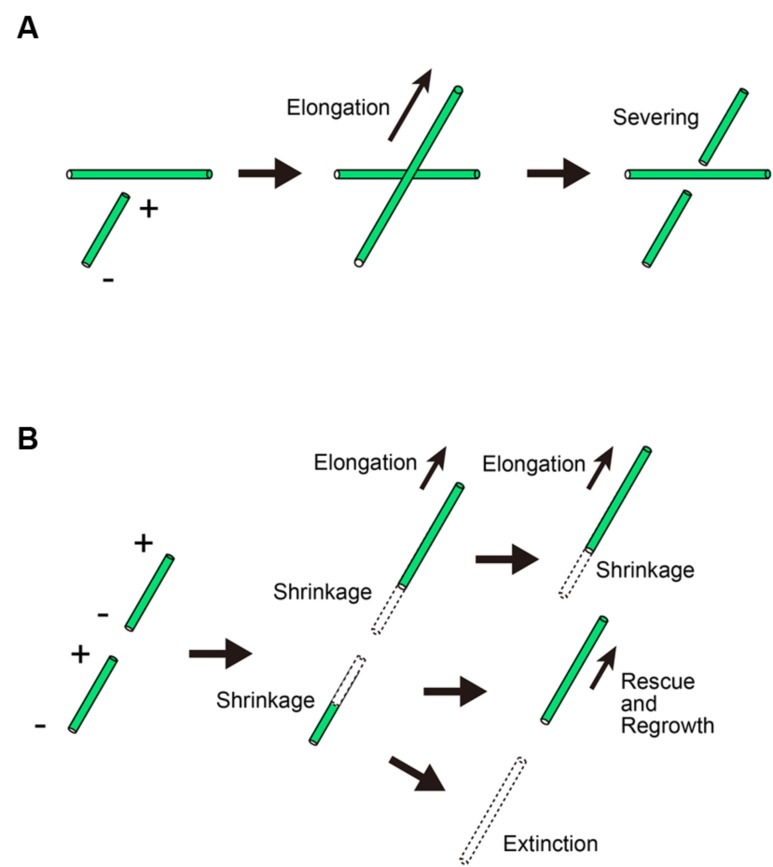
**Microtubule behavior after crossing in the cortical array. (A)** When a microtubule crosses an existing microtubule, the crossing microtubule but not the existing microtubule is severed. + and – denote the plus and minus ends of a microtubule, respectively. **(B)** The severed microtubule depolymerizes at the newly created ends. In some cases, the plus end starts to grow by a rescue event.

Microtubule crossovers made by wide-angle crossing are also severed (**Figure 2A**; Wightman and Turner, 2007). Katanin is recruited to microtubule crossover sites, where it severs the newly crossed but not existing microtubules (Zhang et al., 2013). Because the microtubule ends formed by severing frequently start to depolymerize, microtubule severing by katanin is considered to have a role in the removal of discordant microtubules. Supporting this idea, *A. thaliana* katanin mutants have net-like microtubule arrays in many cell types (Burk and Ye, 2002). In pavement cells, the suppression of katanin recruitment by another microtubule-interacting protein, SPIRAL2, leads to the formation of net-like microtubule arrays (Wightman et al., 2013). However, microtubule severing does not always remove discordant microtubules. The role of katanin in microtubule organization depends on the behavior of microtubule ends after severing. At first, the severed plus ends are thought to depolymerize. If the shrinking microtubules are rescued, however, severing results in the production of new microtubules, rather than the total loss of the severed halves of microtubules (**Figure 2B**; Roll-Mecak and Vale, 2006). In fact, microtubule proliferation by katanin-induced severing is reported to occur in blue-light-induced microtubule reorientation in *A. thaliana* (Lindeboom et al., 2013). The mechanism by which shrinking microtubules are rescued in living cells should be examined in future studies. In *in vitro*-reconstituted microtubules, rescue events likely occur either at the GTP–tubulin patch (Dimitrov et al., 2008) or at the sites of MAP localization on the microtubules (Ichihara et al., 2001). Testing these possibilities would provide insight into the mechanism by which shrinking microtubules are rescued.

## MECHANISM BY WHICH MICROTUBULE ARRAYS ARE ORIENTED IN SPECIFIC DIRECTIONS

The orientation of microtubules in the cortical array is important for plant morphogenesis, because it determines the direction in which individual cells grow. Computer simulation demonstrated that the induction of catastrophe by end walls could orient the cortical array transverse to the cell axis (Eren et al., 2010). Boundary-induced catastrophe was found to occur in light-grown *Arabidopsis* tissues, and the microtubule-binding protein CLASP was shown to suppress edge effects (i.e., the catastrophe events triggered when growing microtubules reach the cell’s edge; Ambrose et al., 2011). CLASP localizes at specific edges, where it suppresses catastrophe induction. As a result, microtubules preferentially pass through the edge where CLASP localizes. Computer simulation showed that modulating the edge effect was sufficient to orient the cortical arrays (Ambrose et al., 2011).

However, later studies showed that CLASP localization in the cell edge is not the only mechanism that modulates cortical array orientation. Cortical arrays change their orientation in response to external (e.g., light and gravity) and internal (e.g., plant hormones) stimuli. When cortical arrays of *A. thaliana* are reoriented from longitudinal to transverse by the simultaneous application of gibberellin and auxin, reorientation starts from the center of the tangential cell surface, leaving longitudinal microtubules around the cell edge (Vineyard et al., 2013). Quantification of the number of growing microtubules using the end-tracking protein EB1 revealed that the number of microtubules generated in the center of the tangential cell surface decreased after hormone application. Therefore, changes in the number of newly formed microtubules toward specific orientations are likely involved in orienting the cortical array. Although microtubule array rotation had been proposed to explain the reorientation of the microtubule array (Takesue and Shibaoka, 1998; Chan et al., 2007), different mechanisms are now known to be involved in this process (Vineyard et al., 2013).

Changes in microtubule formation in blue light-induced microtubule reorientation have been extensively analyzed (Lindeboom et al., 2013). When dark-grown *A. thaliana* seedlings are irradiated with blue light, microtubule arrays reorient from transverse to longitudinal. Microtubule-severing activity at the microtubule crossovers transiently increases in response to blue light treatment, and severing-mediated microtubule proliferation increases the abundance of longitudinal microtubules, such that transverse microtubules are ultimately replaced with longitudinal ones. Microtubule nucleation on existing microtubules is suppressed soon after blue light irradiation, and this suppression is thought to inhibit further reorientation of microtubules (Lindeboom et al., 2013). Observations of a loss-of-function mutant of the *TON2* gene, which encodes a subunit of phosphatase 2A, suggest that microtubule nucleation at a 40-degree angle is important for microtubule reorientation. Together with TON1 (Spinner et al., 2013), which has sequence similarity with the animal centrosome proteins FOP and OFD1 (Azimzadeh et al., 2008), TON2 forms a phosphatase 2A complex. In the *TON2* mutant, the frequency of 40-degree nucleation events among the total number of nucleation events is reduced and blue light-induced reorientation of microtubules is inhibited (Kirik et al., 2012).

Cortical microtubule arrays often take on an oblique orientation (Lloyd and Chan, 2002). Microtubules formed at oblique angles result in stem twisting in *A. thaliana* (Thitamadee et al., 2002). Mutants of tubulin subunits, including those that suppress the GTP hydrolysis of tubulin dimers, have oblique arrays, but the mechanism by which these arrays are oriented is unknown (Ishida et al., 2007). Computer simulation revealed that modifying microtubule dynamics alone would not reproduce microtubule arrays in the oblique orientation (Eren et al., 2010). Different frequencies of branched microtubule nucleation between the left and right sides of microtubules or switching of nucleation patterns from random nucleation to 40-degree branch nucleation are needed to reproduce arrays in the oblique orientation. However, such phenomena have not been demonstrated in living cells. It should be noted that the epidermal cells of *Arabidopsis* seedlings do not form hollow cylinders, and that the orientation of cortical arrays on the outer and inner tangential walls is different (Crowell et al., 2011). Computer simulations are unable to incorporate such complexities of cellular structure. Detailed live imaging of microtubules in tubulin mutants at all faces of a cell and comparisons of microtubule dynamics among the faces of tubulin mutants may provide insight into the mechanism governing the formation of oblique microtubule arrays.

## HOW DOES THE PHRAGMOPLAST EXPAND?

While the mechanisms of cortical array organization are relatively well understood, those in other microtubule arrays, including the preprophase band, mitotic spindle, and phragmoplast, are not. Complex three-dimensional array organization is one of the reasons why such structures are challenging to analyze. Another reason is that the growth and shrinkage of individual microtubules cannot be directly observed in these arrays, except in the early stage of preprophase band development (Dhonukshe and Gadella, 2003). In the case of phragmoplasts, however, photobleaching methods have been successfully applied to analyze microtubule dynamics (Hush et al., 1994; Smertenko et al., 2011; Murata et al., 2013).

The phragmoplast consists of two sets of disk- or toroidal-shaped microtubule arrays, both of which face the newly developing cell plate (Wasteneys, 2002). Some of the microtubules are interdigitated at the phragmoplast equator (Ho et al., 2011), although interdigitated microtubules were not detected in an earlier analysis of the same type of cells (i.e., *Arabidopsis* root cells) by freeze-substitution electron microscopy (Austin et al., 2005). The polarity of microtubules in an array has been analyzed by tubulin decoration methods using lysed cells, and plus ends were found to be located near the site of the cell plate (Euteneuer and McIntosh, 1980). Hush et al. (1994) used fluorescence recovery after photobleaching to demonstrate that microtubules in the phragmoplast turn over at a similar rate as those in interphase cortical arrays. However, it was unclear how and where microtubules were nucleated in the array. Smertenko et al. (2011) proposed that the phragmoplast consists of short microtubules that are nucleated on existing microtubules, based on a computer simulation and on the observed pattern of microtubule recovery after photobleaching. However, the proposed model is inconsistent with previous observations of fixed cells, which revealed that phragmoplasts contain microtubule bundles with partial interdigitation in the midzone, where the cell plate develops (Ho et al., 2011). Recently, we demonstrated that the phragmoplast contains two distinct populations of microtubules, i.e., dynamic microtubules and stable bundles (Murata et al., 2013). The dynamics of these populations are significantly different. The dynamic microtubules are nucleated from stable bundles via γ-tubulin complexes located on the stable bundles. This finding explains both the new model proposed by Smertenko et al. (2011) and previous observations (Ho et al., 2011). The dynamic microtubules correspond to the short microtubules of the new model, and the stable bundles correspond to the interdigitated microtubule bundles observed in fixed cells.

Microtubule turnover in the phragmoplast is likely the driving force of cell plate expansion toward the plasma membrane. When dividing cells are treated with the microtubule depolymerization inhibitor taxol, the rate of cell plate expansion is greatly reduced (Yasuhara et al., 1993). This effect is thought to occur because tubulin heterodimers supplied by depolymerizing microtubules assemble on the outer margin of the phragmoplast, and the addition of microtubules on the outer margin drives phragmoplast expansion, which results in the addition of new cell plate to the margin of the developing cell plate (Jurgens, 2005). However, studies of fluorescent tubulin recovery after photobleaching revealed that microtubules polymerize and depolymerize in the same region of the phragmoplast (Hush et al., 1994). Indeed, microtubule elongation in all regions of the phragmoplast has been demonstrated using GFP-EB1 (Smertenko et al., 2011), the microtubule-plus-end tracking protein. Therefore, the notion that microtubules depolymerize at the inner region of the phragmoplast and the resulting free tubulin molecules polymerize at the outer region is an over-simplified explanation of phragmoplast expansion. Quantitative analyses of local microtubule polymerization and depolymerization are needed to understand this mechanism. We quantified the microtubule depolymerization rate at various points within the phragmoplast using fluorescence loss in photobleaching (FLIP), and found that there was a gradient of microtubule depolymerization rates from the outside to the inside of the phragmoplast. Therefore, differential rates of microtubule depolymerization coupled with uniform polymerization drive the redistribution of the phragmoplast microtubule array (Murata et al., 2013).

## POSSIBLE MECHANISMS OF MICROTUBULE DEPOLYMERIZATION IN THE PHRAGMOPLAST

Because biased depolymerization of microtubules is essential for the progression of cytokinesis, it is important to understand the mechanism of microtubule depolymerization in the phragmoplast. It has been demonstrated that nascent dynamic microtubules are converted to stable bundles around the outer margin of the phragmoplast, and that microtubules in the bundles are preferentially depolymerized in the inner margin (Murata et al., 2013). Microtubules in the stable bundles are likely destabilized at the inner margin, but the molecular mechanism is unknown. It is possible that a MAP kinase cascade is involved in the regulation of microtubule stability in the phragmoplast. A MAP kinase kinase kinase (MAPKKK) NPK1, which is bound to NACK1 kinesin, localizes in the midzone of the phragmoplast and activates downstream kinases of the cascade (Nishihama et al., 2001). Because expression of a dominant negative mutant of NACK1 arrests phragmoplast expansion, phosphorylation by the downstream kinases is likely essential for phragmoplast expansion (Nishihama et al., 2002). In the dominant-negative mutant expressing cells, microtubules in the central region of the phragmoplast persisted for a longer period, suggesting that the phosphorylation pathway is involved in microtubule turnover. A microtubule-bundling protein MAP65-1 is known to be phosphorylated by the downstream MAPK NRK1 (Sasabe et al., 2006). Because overexpression of an NRK1-unphosphorylatable mutant of MAP65-1 stabilizes microtubules in cortical arrays and inhibits phragmoplast expansion (Sasabe et al., 2006), this protein is a good candidate for a regulator of microtubule stability in the bundles. However, it is still unknown how phosphorylation of MAP65-1 causes a gradient of microtubule stability, if this protein plays a role in biased microtubule depolymerization, and whether MAP65-1 is the only target of the MAP kinase cascade.

Microtubules in the phragmoplast seem to be depolymerized by a MAPK-independent mechanism. If NACK1 and MAP kinase cascade proteins depolymerize microtubules, microtubules would depolymerize from their plus ends at the midzone, where the NACK1 and MAP kinase cascade proteins localize. However, microtubules likely depolymerize from their minus ends. In mutants of the microtubule-severing protein katanin, phragmoplast microtubules are long and connected with the nuclear envelope, where minus ends are mainly localized in the early stages of phragmoplast development (Panteris et al., 2011). In addition, GFP-katanin is predominantly localized to the distal region of the phragmoplast (Panteris et al., 2011). Taken together, these findings suggest that katanin-dependent microtubule depolymerization from the minus ends could be involved in microtubule depolymerization in the phragmoplast.

## CONCLUDING REMARKS

In this review, we summarize the structural basis of microtubule dynamics and discuss microtubule organization as a function of microtubule dynamics. However, there is still a large gap in our understanding of how microtubule dynamics documented *in vitro* studies relate to the microtubule conformations observed in living plants cells. Although many microtubule-binding proteins have been characterized in plants, it is largely unknown how these proteins spatially and temporally modulate microtubule dynamics in living cells. Future studies should examine the role of microtubule-binding proteins in the spatial regulation of microtubule dynamics. Particularly, we have almost no information on how minus-end dynamics are regulated in living cells. Newly created minus ends do not depolymerize *in vitro*, but start shrinking *in vivo*. In mammalian cells, the proteins CAMSAP2 and CAMSAP3/Nezha, which regulate minus-end dynamics, have been characterized (Jiang et al., 2014). However, these proteins are not conserved in other organisms (Baines et al., 2009; Jiang et al., 2014). Therefore, a primary focus of future work in this field should be to identify and characterize the proteins that regulate minus-end microtubule dynamics in plant cells.

## Conflict of Interest Statement

The authors declare that the research was conducted in the absence of any commercial or financial relationships that could be construed as a potential conflict of interest.

## References

[B1] Al-Bassam J. Chang F. (2011). Regulation of microtubule dynamics by TOG-domain proteins XMAP215/Dis1 and CLASP. *Trends Cell Biol.* 21 604–614 10.1016/j.tcb.2011.06.007 21782439 PMC3202638

[B2] Al-Bassam J. Kim H. Brouhard G. van Oijen A. Harrison S. C. Chang F. (2010). CLASP promotes microtubule rescue by recruiting tubulin dimers to the microtubule. *Dev. Cell* 19 245–258 10.1016/j.devcel.2010.07.016 20708587 PMC3156696

[B3] Allard J. F. Wasteneys G. O. Cytrynbaum E. N. (2010). Mechanisms of self-organization of cortical microtubules in plants revealed by computational simulations. *Mol. Biol. Cell* 2 278–286 10.1091/mbc.E09-07-0579 19910489 PMC2808237

[B4] Ambrose C. Allard J. F. Cytrynbaum E. N. Wasteneys G. O. (2011). A CLASP-modulated cell edge barrier mechanism drives cell-wide cortical microtubule organization in *Arabidopsis*. *Nat. Commun.* 2:430 10.1038/ncomms1444 PMC3265373 21847104

[B5] Austin J. R. Segui-Simarro J. M. Staehelin L. A. (2005). Quantitative analysis of changes in spatial distribution and plus-end geometry of microtubules involved in plant-cell cytokinesis. *J. Cell Sci.* 118 3895–3903 10.1242/jcs.02512 16105878

[B6] Azimzadeh J. Nacry P. Christodoulidou A. Drevensek S. Camilleri C. Amiour N. (2008). *Arabidopsis* TONNEAU1 proteins are essential for preprophase band formation and interact with centrin. *Plant Cell* 20 2146–2159 10.1105/tpc.107.056812 18757558 PMC2553619

[B7] Baines A. J. Bignone P. A. King M. D. Maggs A. M. Bennett P. M. Pinder J. C. (2009). The CKK domain (DUF1781) binds microtubules and defines the CAMSAP/ssp4 family of animal proteins. *Mol. Biol. Evol.* 26 2005–2014 10.1093/molbev/msp115 19508979

[B8] Bannigan A. Scheible W. R. Lukowitz W. Fagerstrom C. Wadsworth P. Somerville C. (2007). A conserved role for kinesin-5 in plant mitosis. *J. Cell Sci.* 120 2819–2827 10.1242/jcs.009506 17652157

[B10] Bayley P. M. Schilstra M. J. Martin S. R. (1990). Microtubule dynamic instability: numerical simulation of microtubule transition properties using a lateral cap model. *J. Cell Sci.* 95 33–48 2351702 10.1242/jcs.95.1.33

[B9] Bray D. (2001). *Cell Movements: From Molecules to Motility*. New York: Garland Publication

[B11] Burk D. H. Ye Z. H. (2002). Alteration of oriented deposition of cellulose microfibrils by mutation of a katanin-like microtubule-severing protein. *Plant Cell* 14 2145–2160 10.1105/tpc.003947 12215512 PMC150762

[B12] Caplow M. Shanks J. (1996). Evidence that a single monolayer tubulin-GTP cap is both necessary and sufficient to stabilize microtubules. *Mol. Biol. Cell* 7 663–675 10.1091/mbc.7.4.663 8730106 PMC275916

[B13] Carlier M. F. Didry D. Pantaloni D. (1987). Microtubule elongation and guanosine 5′-triphosphate hydrolysis. Role of guanine nucleotides in microtubule dynamics. *Biochemistry* 26 4428–4437 10.1021/bi00388a036 3663597

[B14] Chan J. Calder G. Fox S. Lloyd C. (2007). Cortical microtubule arrays undergo rotary movements in *Arabidopsis* hypocotyl epidermal cells. *Nat. Cell Biol.* 9 171–175 10.1038/ncb1533 17220881

[B15] Chan J. Sambade A. Calder G. Lloyd C. (2009). *Arabidopsis* cortical microtubules are initiated along, as well as branching from, existing microtubules. *Plant Cell* 21 2298–2306 10.1105/tpc.109.069716 19706794 PMC2751946

[B16] Chang-Jie J. Sonobe S. (1993). Identification and preliminary characterization of a 65 kDa higher-plant microtubule-associated protein. *J. Cell Sci.* 105 891–901 8227211 10.1242/jcs.105.4.891

[B17] Chretien D. Fuller S. D. Karsenti E. (1995). Structure of growing microtubule ends: two-dimensional sheets close into tubes at variable rates. *J. Cell Biol.* 129 1311–1328 10.1083/jcb.129.5.1311 7775577 PMC2120473

[B18] Cote R. H. Borisy G. G. (1981). Head-to-tail polymerization of microtubules in vitro. *J. Mol. Biol.* 150 577–599 10.1016/0022-2836(81)90382-X 7328646

[B19] Crowell E. F. Timpano H. Desprez T. Franssen-Verheijen T. Emons A. M. Hofte H. (2011). Differential regulation of cellulose orientation at the Inner and outer face of epidermal cells in the *Arabidopsis* hypocotyl. *Plant Cell* 23 2592–2605 10.1105/tpc.111.087338 21742992 PMC3226210

[B20] Dhonukshe P. Gadella T. W. Jr. (2003). Alteration of microtubule dynamic instability during preprophase band formation revealed by yellow fluorescent protein-CLIP170 microtubule plus-end labeling. *Plant Cell* 15 597–611 10.1105/tpc.008961 12615935 PMC150016

[B21] Dimitrov A. Quesnoit M. Moutel S. Cantaloube I. Pous C. Perez F. (2008). Detection of GTP-tubulin conformation *in vivo* reveals a role for GTP remnants in microtubule rescues. *Science* 322 1353–1356 10.1126/science.1165401 18927356

[B22] Dixit R. Cyr R. (2004). Encounters between dynamic cortical microtubules promote ordering of the cortical array through angle-dependent modifications of microtubule behavior. *Plant Cell* 16 3274–3284 10.1105/tpc.104.026930 15539470 PMC535873

[B23] Drechsel D. N. Kirschner M. W. (1994). The minimum GTP cap required to stabilize microtubules. *Curr. Biol.* 4 1053–1061 10.1016/S0960-9822(00)00243-8 7704569

[B24] Dustin P. (1984). *Microtubules.* New York: Springer 10.1007/978-3-642-69652-7

[B25] Eren E. C. Dixit R. Gautam N. (2010). A three-dimensional computer simulation model reveals the mechanisms for self-organization of plant cortical microtubules into oblique arrays. *Mol. Biol. Cell* 21 2674–2684 10.1091/mbc.E10-02-0136 20519434 PMC2912353

[B26] Euteneuer U. McIntosh J. R. (1980). Polarity of midbody and phragmoplast microtubules. *J. Cell Biol.* 87 509–515 10.1083/jcb.87.2.509 7430255 PMC2110749

[B27] Evans L. Mitchison T. Kirschner M. (1985). Influence of the centrosome on the structure of nucleated microtubules. *J. Cell Biol.* 100 1185–1191 10.1083/jcb.100.4.1185 4038981 PMC2113747

[B28] Gardiner J. (2013). The evolution and diversification of plant microtubule-associated proteins. *Plant J.* 75 219–229 10.1111/tpj.12189 23551562

[B29] Gardner M. K. Hunt A. J. Goodson H. V. Odde D. J. (2008). Microtubule assembly dynamics: new insights at the nanoscale. *Curr. Opin. Cell Biol.* 20 64–70 10.1016/j.ceb.2007.12.003 18243676 PMC2547410

[B30] Gupta M. L. Jr. Carvalho P. Roof D. M. Pellman D. (2006). Plus end-specific depolymerase activity of Kip3 a kinesin-8 protein, explains its role in positioning the yeast mitotic spindle. *Nat. Cell Biol.* 8 913–923 10.1038/ncb1457 16906148

[B31] Hamada T. (2007). Microtubule-associated proteins in higher plants. *J. Plant Res.* 120 79–98 10.1007/s10265-006-0057-9 17285404

[B32] Hamada T. (2014). Lessons from in vitro reconstitution analyses of plant microtubule-associated proteins. *Front. Plant. Sci.* 5:409 10.3389/fpls.2014.00409 PMC4141329 25202315

[B33] Ho C. M. Hotta T. Guo F. Roberson R. W. Lee Y. R. Liu B. (2011). Interaction of antiparallel microtubules in the phragmoplast is mediated by the microtubule-associated protein MAP65-3 in *Arabidopsis*. *Plant Cell* 23 2909–2923 10.1105/tpc.110.078204 21873565 PMC3180800

[B34] Horio T. Hotani H. (1986). Visualization of the dynamic instability of individual microtubules by dark-field microscopy. *Nature* 321 605–607 10.1038/321605a0 3713844

[B35] Hush J. M. Wadsworth P. Callaham D. A. Hepler P. K. (1994). Quantification of microtubule dynamics in living plant cells using fluorescence redistribution after photobleaching. *J. Cell Sci.* 107 775–784 8056836 10.1242/jcs.107.4.775

[B36] Hussey P. J. Hawkins T. J. Igarashi H. Kaloriti D. Smertenko A. (2002). The plant cytoskeleton: recent advances in the study of the plant microtubule-associated proteins MAP-65, MAP-190 and the *Xenopus* MAP215-like protein, MOR1. *Plant Mol. Biol.* 50 915–924 10.1023/A:1021236307508 12516862

[B37] Ichihara K. Kitazawa H. Iguchi Y. Hotani H. Itoh T. J. (2001). Visualization of the stop of microtubule depolymerization that occurs at the high-density region of microtubule-associated protein 2 (MAP2). *J. Mol. Biol.* 312 107–118 10.1006/jmbi.2001.4934 11545589

[B38] Ishida T. Kaneko Y. Iwano M. Hashimoto T. (2007). Helical microtubule arrays in a collection of twisting tubulin mutants of *Arabidopsis thaliana*. *Proc. Natl. Acad. Sci. U.S.A.* 104 8544–8549 10.1073/pnas.0701224104 17488810 PMC1895986

[B39] Jiang K. Hua S. S. Mohan R. Grigoriev I. Yau K. W. Liu Q. Y. (2014). Microtubule minus-end stabilization by polymerization-driven CAMSAP deposition. *Dev. Cell* 28 295–309 10.1016/j.devcel.2014.01.001 24486153

[B40] Job D. Valiron O. Oakley B. (2003). Microtubule nucleation. *Curr. Opin. Cell Biol.* 15 111–117 10.1016/S0955-0674(02)00003-0 12517712

[B41] Jurgens G. (2005). Cytokinesis in higher plants. *Annu. Rev. Plant Biol.* 56 281–299 10.1146/annurev.arplant.55.031903.141636 15862097

[B42] Kawamura E. Wasteneys G. O. (2008). MOR1 the *Arabidopsis thaliana* homologue of *Xenopus* MAP215 promotes rapid growth and shrinkage, and suppresses the pausing of microtubules *in vivo*. *J. Cell Sci.* 121 4114–4123 10.1242/jcs.039065 19033380

[B43] Kerssemakers J. W. Munteanu E. L. Laan L. Noetzel T. L. Janson M. E. Dogterom M. (2006). Assembly dynamics of microtubules at molecular resolution. *Nature* 442 709–712 10.1038/nature04928 16799566

[B44] Kirik A. Ehrhardt D. W. Kirik V. (2012). TONNEAU2/FASS regulates the geometry of microtubule nucleation and cortical array organization in interphase *Arabidopsis* cells. *Plant Cell* 24 1158–1170 10.1105/tpc.111.094367 22395485 PMC3336111

[B45] Kirschner M. Mitchison T. (1986). Beyond self-assembly: from microtubules to morphogenesis. *Cell* 45 329–342 10.1016/0092-8674(86)90318-1 3516413

[B46] Kollman J. M. Merdes A. Mourey L. Agard D. A. (2011). Microtubule nucleation by gamma-tubulin complexes. *Nat. Rev. Mol. Cell Biol.* 12 709–721 10.1038/nrm3209 21993292 PMC7183383

[B47] Komaki S. Abe T. Coutuer S. Inze D. Russinova E. Hashimoto T. (2010). Nuclear-localized subtype of end-binding 1 protein regulates spindle organization in *Arabidopsis*. *J. Cell Sci.* 123 451–459 10.1242/jcs.062703 20067996

[B48] Kumar P. Wittmann T. (2012). +TIPs: SxIPping along microtubule ends. *Trends Cell Biol.* 22 418–428 10.1016/j.tcb.2012.05.005 22748381 PMC3408562

[B49] Lindeboom J. J. Nakamura M. Hibbel A. Shundyak K. Gutierrez R. Ketelaar T. (2013). A mechanism for reorientation of cortical microtubule arrays driven by microtubule severing. *Science* 342 1245533 10.1126/science.1245533 24200811

[B50] Lloyd C. Chan J. (2002). Helical microtubule arrays and spiral growth. *Plant Cell* 14 2319–2324 10.1105/tpc.141030 12368488 PMC543213

[B51] Lowe J. Li H. Downing K. H. Nogales E. (2001). Refined structure of alpha beta-tubulin at 3.5 A resolution. *J. Mol. Biol.* 313 1045–1057 10.1006/jmbi.2001.5077 11700061

[B52] Lucas J. R. Courtney S. Hassfurder M. Dhingra S. Bryant A. Shaw S. L. (2011). Microtubule-associated proteins MAP65-1 and MAP65-2 positively regulate axial cell growth in etiolated *Arabidopsis* hypocotyls. *Plant Cell* 23 1889–1903 10.1105/tpc.111.084970 21551389 PMC3123956

[B53] Mandelkow E. M. Mandelkow E. Milligan R. A. (1991). Microtubule dynamics and microtubule caps: a time-resolved cryo-electron microscopy study. *J. Cell Biol.* 114 977–991 10.1083/jcb.114.5.977 1874792 PMC2289108

[B54] Mayr M. I. Hummer S. Bormann J. Gruner T. Adio S. Woehlke G. (2007). The human kinesin Kif18A is a motile microtubule depolymerase essential for chromosome congression. *Curr. Biol.* 17 488–498 10.1016/j.cub.2007.02.036 17346968

[B55] Miki T. Naito H. Nishina M. Goshima G. (2014). Endogenous localizome identifies 43 mitotic kinesins in a plant cell. *Proc. Natl. Acad. Sci. U.S.A.* 111 E1053–E1061 10.1073/pnas.1311243111 24591632 PMC3964101

[B56] Mitchison T. Kirschner M. (1984a). Dynamic instability of microtubule growth. *Nature* 312 237–242 10.1038/312237a0 6504138

[B57] Mitchison T. Kirschner M. (1984b). Microtubule assembly nucleated by isolated centrosomes. *Nature* 312 232–237 10.1038/312232a0 6504137

[B58] Mitchison T. J. (1993). Localization of an exchangeable GTP binding site at the plus end of microtubules. *Science* 261 1044–1047 10.1126/science.8102497 8102497

[B59] Moores C. A. Yu M. Guo J. Beraud C. Sakowicz R. Milligan R. A. (2002). A mechanism for microtubule depolymerization by KinI kinesins. *Mol. Cell.* 9 903–909 10.1016/S1097-2765(02)00503-8 11983180

[B60] Murata T. Sano T. Sasabe M. Nonaka S. Higashiyama T. Hasezawa S. (2013). Mechanism of microtubule array expansion in the cytokinetic phragmoplast. *Nat. Commun.* 4 1967 10.1038/ncomms2967 PMC3709505 23770826

[B61] Murata T. Sonobe S. Baskin T. I. Hyodo S. Hasezawa S. Nagata T. (2005). Microtubule-dependent microtubule nucleation based on recruitment of γ-tubulin in higher plants. *Nat. Cell Biol.* 7 961–968 10.1038/ncb1306 16138083

[B62] Murata T. Tanahashi T. Nishiyama T. Yamaguchi K. Hasebe M. (2007). How do plants organize microtubules without a centrosome? *J. Integr. Plant Biol.* 49 1154–1163 10.1111/j.1672-9072.2007.00545.x

[B63] Nakamura M. Ehrhardt D. W. Hashimoto T. (2010). Microtubule and katanin-dependent dynamics of microtubule nucleation complexes in the acentrosomal *Arabidopsis* cortical array. *Nat. Cell Biol.* 12 1064–1070 10.1038/ncb2110 20935636

[B64] Newton C. N. Wagenbach M. Ovechkina Y. Wordeman L. Wilson L. (2004). MCAK, a Kin I kinesin, increases the catastrophe frequency of steady-state HeLa cell microtubules in an ATP-dependent manner *in vitro*. *FEBS Lett.* 572 80–84 10.1016/j.febslet.2004.06.093 15304328

[B65] Nishihama R. Ishikawa M. Araki S. Soyano T. Asada T. Machida Y. (2001). The NPK1 mitogen-activated protein kinase kinase kinase is a regulator of cell-plate formation in plant cytokinesis. *Genes Dev.* 15 352–363 10.1101/gad.863701 11159915 PMC312623

[B66] Nishihama R. Soyano T. Ishikawa M. Araki S. Tanaka H. Asada T. (2002). Expansion of the cell plate in plant cytokinesis requires a kinesin-like protein/MAPKKK complex. *Cell* 109 87–99 10.1016/S0092-8674(02)00691-8 11955449

[B67] Nogales E. Wang H. W. (2006). Structural mechanisms underlying nucleotide-dependent self-assembly of tubulin and its relatives. *Curr. Opin. Struct. Biol.* 16 221–229 10.1016/j.sbi.2006.03.005 16549346

[B68] Nogales E. Wolf S. G. Downing K. H. (1998). Structure of the alpha beta tubulin dimer by electron crystallography. *Nature* 391 199–203 10.1038/34465 9428769

[B69] O’Brien E. T. Voter W. A. Erickson H. P. (1987). GTP hydrolysis during microtubule assembly. *Biochemistry* 26 4148–4156 10.1021/bi00387a061 3651443

[B70] Oda Y. Fukuda H. (2012). Initiation of cell wall pattern by a Rho- and microtubule-driven symmetry breaking. *Science* 337 1333–1336 10.1126/science.1222597 22984069

[B71] Panteris E. Adamakis I. D. S. Voulgari G. Papadopoulou G. (2011). A role for katanin in plant cell division: microtubule organization in dividing root cells of fra2 and lue1 *Arabidopsis thaliana* mutants. *Cytoskeleton* 68 401–413 10.1002/cm.20522 21721142

[B72] Portran D. Zoccoler M. Gaillard J. Stoppin-Mellet V. Neumann E. Arnal I. (2013). MAP65/Ase1 promte microtubule flexibility. *Mol. Biol. Cell* 24 1964–1973 10.1091/mbc.E13-03-0141 23615441 PMC3681700

[B73] Roll-Mecak A. Vale R. D. (2006). Making more microtubules by severing: a common theme of noncentrosomal microtubule arrays? *J. Cell Biol.* 175 849–851 10.1083/jcb.200611149 17178905 PMC2064694

[B74] Sasabe M. Soyano T. Takahashi Y. Sonobe S. Igarashi H. Itoh T. J. (2006). Phosphorylation of NtMAP65-1 by a MAP kinase down-regulates its activity of microtubule bundling and stimulates progression of cytokinesis of tobacco cells. *Genes Dev.* 20 1004–1014 10.1101/gad.1408106 16598040 PMC1472297

[B75] Schek H. T. 3rd Gardner M. K. Cheng J. Odde D. J. Hunt A. J. (2007). Microtubule assembly dynamics at the nanoscale. *Curr. Biol.* 17 1445–1455 10.1016/j.cub.2007.07.011 17683936 PMC2094715

[B76] Shaw S. L. Kamyar R. Ehrhardt D. W. (2003). Sustained microtubule treadmilling in *Arabidopsis* cortical arrays. *Science* 300 1715–1718 10.1126/science.1083529 12714675

[B77] Smertenko A. Saleh N. Igarashi H. Mori H. Hauser-Hahn I. Jiang C. J. (2000). A new class of microtubule-associated proteins in plants. *Nat. Cell Biol.* 2 750–753 10.1038/35036390 11025667

[B78] Smertenko A. P. Piette B. Hussey P. J. (2011). The origin of phragmoplast asymmetry. *Curr. Biol.* 21 1924–1930 10.1016/j.cub.2011.10.012 22079114

[B79] Spinner L. Gadeyne A. Belcram K. Goussot M. Moison M. Duroc Y. (2013). A protein phosphatase 2A complex spatially controls plant cell division. *Nat. Commun.* 4 1863 10.1038/ncomms2831 23673648

[B80] Stoppin-Mellet V. Fache V. Portran D. Martiel J. L. Vantard M. (2013). MAP65 coordinate microtubule growth during bundle formation. *PloS one* 8:e56808 10.1371/journal.pone.0056808 PMC3578873 23437247

[B81] Takesue K. Shibaoka H. (1998). The cyclic reorientation of cortical microtubules in epidermal cells of azuki bean epicotyls: the role of actin filaments in the progression of the cycle. *Planta* 205 539–546 10.1007/s004250050353 9684358

[B82] Thitamadee S. Tuchihara K. Hashimoto T. (2002). Microtubule basis for left-handed helical growth in *Arabidopsis*. *Nature* 417 193–196 10.1038/417193a 12000963

[B83] Tran P. T. Joshi P. Salmon E. D. (1997). How tubulin subunits are lost from the shortening ends of microtubules. *J. Struct. Biol.* 118 107–118 10.1006/jsbi.1997.3844 9126637

[B84] Tulin A. McClerklin S. Huang Y. Dixit R. (2012). Single-molecule analysis of the microtubule cross-linking protein MAP65-1 reveals a molecular mechanism for contact-angle-dependent microtubule bundling. *Biophys. J.* 102 802–809 10.1016/j.bpj.2012.01.008 22385851 PMC3283812

[B85] van der Vaart B. Akhmanova A. Straube A. (2009). Regulation of microtubule dynamic instability. *Biochem. Soc. Trans.* 37 1007–1013 10.1042/BST0371007 19754441

[B86] Varga V. Helenius J. Tanaka K. Hyman A. A. Tanaka T. U. Howard J. (2006). Yeast kinesin-8 depolymerizes microtubules in a length-dependent manner. *Nat. Cell Biol.* 8 957–962 10.1038/ncb1462 16906145

[B87] Vineyard L. Elliott A. Dhingra S. Lucas J. R. Shaw S. L. (2013). Progressive transverse microtubule array organization in hormone-induced *Arabidopsis* hypocotyl cells. *Plant Cell* 25 662–676 10.1105/tpc.112.107326 23444330 PMC3608785

[B88] Walker R. A. O’Brien E. T. Pryer N. K. Soboeiro M. F. Voter W. A. Erickson H. P. (1988). Dynamic instability of individual microtubules analyzed by video light microscopy: rate constants and transition frequencies. *J. Cell Biol.* 107 1437–1448 10.1083/jcb.107.4.1437 3170635 PMC2115242

[B89] Wang H. W. Nogales E. (2005). Nucleotide-dependent bending flexibility of tubulin regulates microtubule assembly. *Nature* 435 911–915 10.1038/nature03606 15959508 PMC1386036

[B90] Wasteneys G. O. (2002). Microtubule organization in the green kingdom: chaos or self-order? *J. Cell Sci*. 115 1345–1354 11896182 10.1242/jcs.115.7.1345

[B91] Whittington A. T. Vugrek O. Wei K. J. Hasenbein N. G. Sugimoto K. Rashbrooke M. C. (2001). MOR1 is essential for organizing cortical microtubules in plants. *Nature* 411 610–613 10.1038/35079128 11385579

[B93] Wiedemeier A. M. Judy-March J. E. Hocart C. H. Wasteneys G. O. Williamson R. E. Baskin T. I. (2002). Mutant alleles of *Arabidopsis* RADIALLY SWOLLEN 4 and 7 reduce growth anisotropy without altering the transverse orientation of cortical microtubules or cellulose microfibrils. *Development* 129 4821–4830 12361973 10.1242/dev.129.20.4821

[B92] Wightman R. Chomicki G. Kumar M. Carr P. Turner S. R. (2013). SPIRAL2 determines plant microtubule organization by modulating microtubule severing. *Curr. Biol.* 23 1902–1907 10.1016/j.cub.2013.07.061 24055158 PMC3793865

[B94] Wightman R. Turner S. R. (2007). Severing at sites of microtubule crossover contributes to microtubule alignment in cortical arrays. *Plant J.* 52 742–751 10.1111/j.1365-313X.2007.03271.x 17877711

[B95] Yao M. Wakamatsu Y. Itoh T. J. Shoji T. Hashimoto T. (2008). *Arabidopsis* SPIRAL2 promotes uninterrupted microtubule growth by suppressing the pause state of microtubule dynamics. *J. Cell Sci.* 121 2372–2381 10.1242/jcs.030221 18577573

[B96] Yasuhara H. Sonobe S. Shibaoka H. (1993). Effects of taxol on the development of the cell plate and of the phragmoplast in tobacco BY-2 cells. *Plant Cell Physiol.* 34 21–29

[B97] Zhang Q. Fishel E. Bertroche T. Dixit R. (2013). Microtubule severing at crossover sites by katanin generates ordered cortical microtubule arrays in *Arabidopsis*. *Curr. Biol.* 23 2191–2195 10.1016/j.cub.2013.09.018 24206847

